# The complete mitochondrial genome of *Aconurella prolixa* (Lethierry 1885) (Hemiptera: Cicadellidae: Deltocephalinae: Chiasmini)

**DOI:** 10.1080/23802359.2021.2008834

**Published:** 2021-12-20

**Authors:** Kaiqi Wu, Minhui Yan, Yongxia Zhang, Christopher H. Dietrich, Yani Duan

**Affiliations:** a Anhui Province Key Laboratory of Integrated Pest Management on Crops, Key Laboratory of Biology and Sustainable Management of Plant Diseases and Pests of Anhui Higher Education Institutes, School of Plant Protection, Anhui Agricultural University, Hefei, Anhui, China; b Illinois Natural History Survey, Prairie Research Institute, University of Illinois, Champaign, IL, USA

**Keywords:** Mitochondrial genome, Cicadellidae, Deltocephalinae, *Aconurella prolixa*, phylogeny

## Abstract

The complete mitochondrial genome of the widespread leafhopper species *Aconurella prolixa* (Hemiptera: Cicadellidae: Deltocephalinae: Chiasmini) was obtained via next-generation sequencing. This mitochondrial genome is 14,832 bp in length with the 37 classical eukaryotic mitochondrial genes and a control region. All 13 protein-coding genes (PCGs) are initiated with ATN, except *ND5* uses TTG as the start codon, and terminate with TAA or TAG with the exception of *COX2* and *ND4* which use a single T residue as the stop codon. Twenty-one of the 22 transfer RNA (tRNAs) genes have the typical clover-leaf structure except for *trnS1*. Unlike some other species of deltocephalinae, no tRNA rearrangements were detected. The monophyly of Cicadellidae and Deltocephalinae, as well as the monophyly of Chiasmini, with a sister relationship between *A. prolixa* and (*Exitianus indicus + Nephotettix cincticeps*) is supported by Bayesian inference phylogenetic analyses based on 13 PCGs.

*Aconurella prolixa* (Lethierry 1885) belongs to the grass-specialist deltocephaline tribe Chiasmini (Hemiptera: Cicadellidae: Deltocephalinae). It is the type species of *Aconurella*, and is widely distributed throughout the tropical and subtropical zones of the Old World. Twenty-five species of *Aconurella* are known but most appear to have relatively narrow distributions (Duan and Zhang 2012). To date, no whole mitogenome sequences were available for *Aconurella* and the mitogenomes of only two other species of Chiasmini have been sequenced. In this study, we sequenced and annotated the complete mitogenome of *A. prolixa* to facilitate a better understanding of the mitochondrial characteristics and the evolutionary history of Cicadellidae. Some species of Deltocephalinae have rearrangements in the order of tRNAs in their mitogenomes but, so far, these have been reported in only two genera belonging to the tribes Macrostelini and Opsiini (Du et al. 2017, 2019).

The sequenced individual was collected from Rongshui Miao Autonomous County, Guangxi, China (109°15'18″E, 25°05'03″N) in July 2019. A voucher specimen is stored at the Insect Systematic Laboratory of Anhui Agricultural University (voucher number GX005, Kaiqi Wu, wukaiqi@ahau.edu.cn). Total genomic DNA was extracted using the cetyltrimethyl ammonium bromide (CTAB) method (Shahjahan et al. 1995). The mitogenome was sequenced on the Illumina NovaSeq platform with 150 bp paired-end reads and yielded 2.42 GB paired raw reads. The quality of data was checked by FastQC (Andrews 2016). A total of 2.10 GB of clean paired-end reads (Phred scores >20) were quality-trimmed and assembled using Geneious 8.1.3 (Kearse et al. 2012) with default parameters and the mitochondrial genome of *Nephotettix cincticeps* (Uhler 1896) (GenBank No: KP749836) used as a reference. Gene annotation was carried out in Geneious 8.1.3 (Kearse et al. 2012). PCGs were determined by open reading frames; rRNAs and tRNAs were identified using MITOS (Bernt et al. 2013).

The complete mitogenome of *A. prolixa* is 14,832 bp in length (GenBank No: MZ433366), containing 13 PCGs, 22 tRNAs, 2 ribosomal RNA genes (rRNAs), and 1 control region. Although rearrangements of tRNAs have been reported in a few Deltocephalinae, the gene order and orientation of *A. prolixa* are identical to that of most other leafhoppers as well as to the model species *Drosophila melanogaster* Meigen 1830. The A + T content of the mitogenome is 74.9% (A = 42.3%, T = 32.6%, G = 10.4%, C = 14.7%) which is significantly biased toward AT. All PCGs started with the canonical putative start codon ATN (4 with ATA, 5 with ATG, 1 with ATC and 1 with ATT) except for the *ND5* which started with TTG instead, and stopped with TAR (3 with TAG, 8 with TAA), except *ND4* and *COX2*, which stopped with single T. There are 22 tRNA genes, ranging from 62 to 71 bp in length. The secondary structure of the 21 tRNAs is a typical clover-leaf structure except for *trnS1*. Among the two rRNA genes, the *16SrRNA* is 1207 bp in length, located between *trnL1* and *trnV*, and the *12SrRNA* is 751 bp in length, located between *trnV* and control region. The control region is located between *12SrRNA* and *trnI*, which is 411 bp in length.

To estimate phylogenetic relationships within Cidadellidae, we used 50 species (including 48 Cicadellidae and 2 outgroups from Cercopoidea and Cicadoidea). The nucleotide sequences of the 13 PCGs were aligned by multiple alignments using the MAFFT 7 plugin in PhyloSuite 1.2.2 (Zhang et al. 2020). Gaps and ambiguous sites were removed using the Gblocks 0.91 b plugin in PhyloSuite 1.2.2 (Zhang et al. 2020). The aligned PCGs data of the different species were concatenated using PhyloSuite 1.2.2 (Zhang et al. 2020) and the best-fit partitioning schemes and models of evolution for phylogenetic analyses were determined using PartitionFinder 2.7 plugin in PhyloSuite 1.2.2 (Zhang et al. 2020) (Table 1). The phylogenetic relationships were reconstructed with the MrBayes plugin in PhyloSuite 1.2.2 (Zhang et al. 2020). The result supports the monophyly of Cicadellidae and Deltocephalinae, as well as the monophyly of Chiasmini, with a sister relationship between *A. prolixa* and (*Exitianus indicus* (Distant 1908)*+N.cincticeps*) with maximum Bayesian posterior probability (1) (Figure 1). Relationships within Deltocephalinae are largely consistent with those found in previous analyses based on Sanger sequencing data and morphology (Zahniser and Dietrich 2013) with differences confined to areas of the tree with low branch support (e.g. relationships among grass-feeding tribes Chiasmini, Macrostelini and Mukariini). Overall branch support in our analysis is higher, suggesting that further analyses of mitogenome sequences can help improve knowledge of the phylogenetic relationships among major lineages of this subfamily. Complete mitogenome sequences for additional Deltocephaline specimens are needed to facilitate broader comparison and to identify features of potential phylogenetic and evolutionary significance.

**Figure 1.**
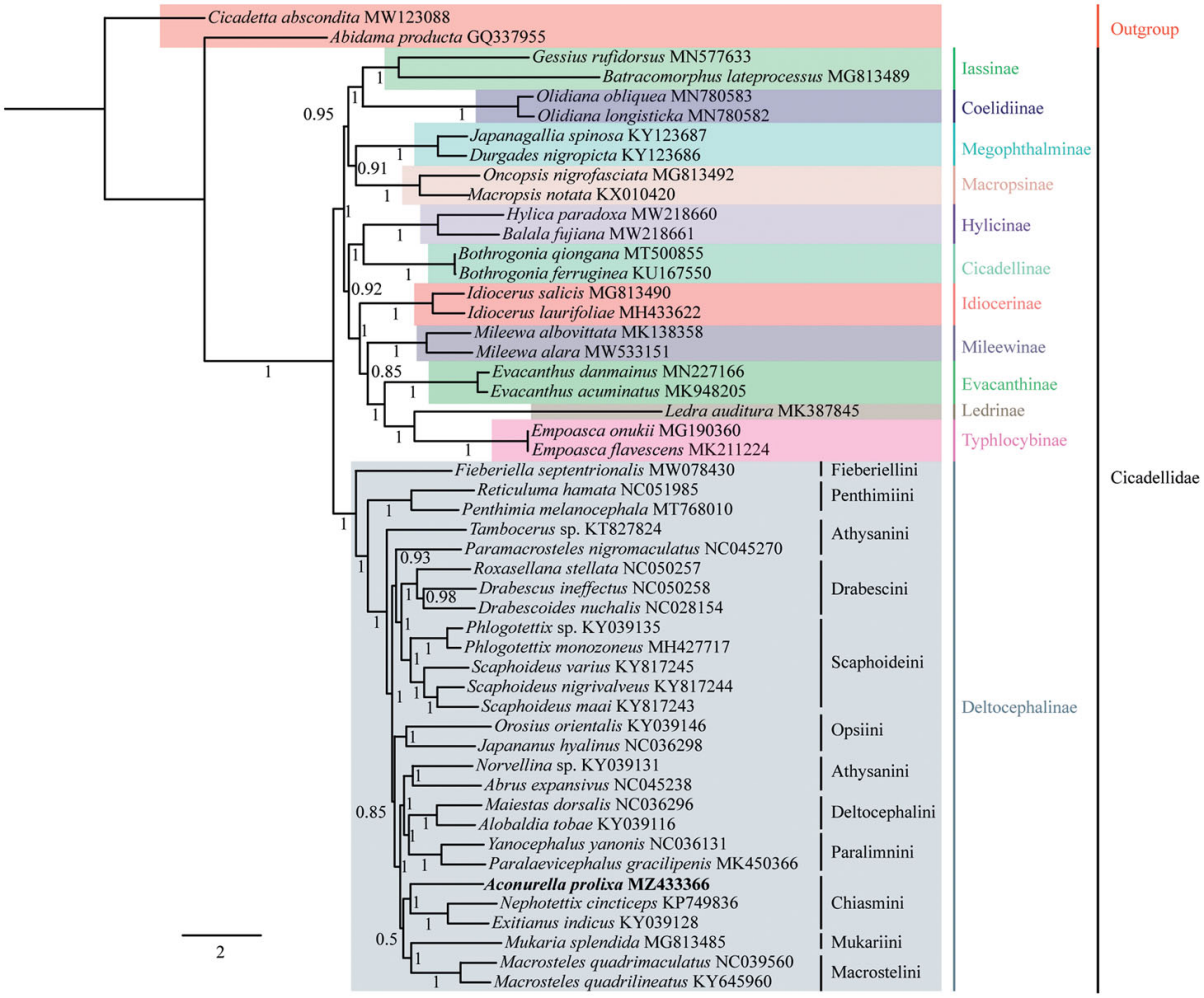
BI consensus tree for Cicadellidae based on concatenated 13 PCGs with branch support shown as Bayesian posterior probabilities. Each species GenBank accession numbers are indicated to the right of the names of the included species.

**Table 1.** Best-fit partitioning schemes and models of evolution determined by PartitionFinder.

Subset Partitions	Model
P1: (*ATP*6_pos1, *ATP*8_pos2, *COX*2_pos1, *COX*3_pos1, *CYTB*_pos1)	GTR + I + G
P2: (*ATP*6_pos2, *COX*1_pos2, *COX*2_pos2, *COX*3_pos2, *CYTB*_pos2)	GTR + I + G
P3: (*COX*1_pos3, *ATP*6_pos3)	HKY + I + G
P4: (*ND*2_pos1, *ND*3_pos1, *ND*6_pos1, *ATP*8_pos1)	GTR + I + G
P5: (*ATP*8_pos3, *CYTB*_pos3, *COX*2_pos3, *COX*3_pos3, *ND3*_pos3, *ND*6_pos3)	GTR + G
P6: (*COX*1_pos1)	GTR + I + G
P7: (*ND*1_pos1, *ND4l*_pos1, *ND*4_pos1, *ND*5_pos1)	GTR + I + G
P8: (*ND*1_pos2, *ND4l*_pos2, *ND*4_pos2, *ND*5_pos2)	GTR + I + G
P9: (*ND*1_pos3, *ND4l*_pos3, *ND*4_pos3, *ND*5_pos3)	GTR + G
P10: (*ND2*_pos2, *ND3*_pos2, *ND6*_pos2)	GTR + G
P11: (*ND2*_pos3)	GTR + G

## Data Availability

The data that support the findings of this study are openly available in NCBI at https://www.ncbi.nlm.nih.gov/nuccore/MZ433366.1/, under GenBank accession number MZ433366. The associated BioProject, SRA, and Bio-Sample numbers are PRJNA754105, SRR15429026, and SAMN20741744, respectively.
